# Ubi Est Morbus?

**Published:** 1894-04-14

**Authors:** 


### The Hospital

An Institutional Journal of

The Medical Sciences and Hospital Administration, with

Vol. XVI.—No. 394.] AN EXTRA NURSING SUPPLEMENT. [April 14, 1894.

### Ubi est Morbus?

There are not too many philosophical thinkers in medicine, nor, indeed, in any other calling. But there are some; and when we discover them we do well to encourage them to speak freely, and to encourage ourselves also in that docility of temper which is the beginning of all knowledge and mental growth. Professor Virchow is one of our seeing and understanding men. There is much to be learnt from him, not, perhaps, now of new knowledge, but of that which is better than mere knowledge, true seeing and comprehensive definiteness of knowledge and thought. Virchow discussed to the members of the International Medical Congress at Rome last week on "Morgagni and Anatomical Thought." The subject may seem to the mere clinician somewhat academical, and he will perhaps pass it by. But Virchow, though a laboratory worker, is the delight of the scientific clinician; and Morgagni, though an anatomist, was an anatomist whose highest ideal was that of supplying an anatomical lantern for the surer guidance of the groping, and, therefore, much-blundering practising doctor of his period. What is disease? That was the question of Galen and the generations which followed him. Surely a question of fundamental importance. Where is disease? This, which was the question of Morgagni, seems, and perhaps is, much simpler. But it is certainly not less fundamental. Perhaps, indeed, it is more so; for if we have not been able to find out "where" a thing is, there would seem to be very small probability of our certifying ourselves as to "what" it is. From the utilitarian standpoint of bedside work, the address of Virchow on "Anatomical Thought" is charged with light and suggestiveness.

Ubi est morbus? Where is the disease? This, Virchow affirms, "is the question to begin with both in examining a living patient and a dead body." Not what? but where? Where, where exactly? And then afterwards, what? The thing is true; and, many will say, quite obvious. It is indeed quite obvious to the thought when placed out so definitely by a man of Virchow's authority. But is it as obvious that the principle is carried out in practice at the bedside of every man who employs a physician of the *fin de siècle?* Is it not but too certain that many a practising doctor never thinks of asking himself "where" the disease is, or even "what" it is? He notes a few manifest symptoms, promptly masses them together and gives them a name, sends in his dose, and, as the conjuror says, "there you are." This is not medical practice; it is "scamping quackery"; and it is one of the most urgent of all the duties of the medical journalist to expose it, to denounce it, and as far as may be to render its continued existence impossible.

There are, Virchow tells us, no such things as "general diseases." Our not very remote predecessors thought there certainly were. "They examined into antecedents, they established a clinical picture of the disease, they determined the structural alterations of the body, and gave the whole a name. In spite of all this the nature of the disease was usually by no means clear; the most arbitrary hypotheses were relied on, and even regarded as scientific. What theories have not been maintained as to the nature of fever and of inflammation? Pathological anatomy teaches us that there is no diseased body which has undergone change in all its parts"! In a word, every disease of every kind known to science is a local disease, at any rate in its inception and origin; and the first business of every practitioner who is a scientific workman in his calling is to find out "where" the disease which is now injuring a part or the whole of the body is actually located in the body, and where its processes commenced. When this is done, the larger half of the diagnosis is already made, and the indications for scientific treatment have begun to dawn upon the mind.

It is the clinician especially, the so-called "practical man," who is most deeply concerned in this primary question of the actual whereabouts of the disease. Virchow says: "Anatomical thought reaches much further than the dominion of pathological anatomy; it is no longer confined to the visible changes exposed by the anatomist's knife, but is concerned also with the vital functions of organs, and so includes a great part of what modern division of labour has given to the clinician." Virchow has, in short, discovered what has long been looming before the mind of the philosophic practitioner, that the division of labour has made the practitioner of general medicine a true specialist.

He is the specialist of specialists, because the broadest and most comprehensive of them all. For him anatomical thought, not the bare knowledge of anatomical facts, but daily anatomical thought, is the most necessary and fundamental of all his manifold knowledges. That the most important of all specialties is common practice, and that that practice needs larger and more varied resources than any other specialty, is quite obvious to the mind of Virchow, as to that of other deep seeing thinkers. The profoundest depths of anatomical and pathological knowledge must often be sounded by the general physician perforce, in even the most elementary of all the parts of his daily duty, the duty of discovering, first of all, exactly "where" the disease is. "The search after the *sedes morbi*," as Virchow insists, "has advanced from the organs to the tissues, and from the tissues to the cells. In practical medicine the principle of local treatment has continually progressed; each year treatment by drugs and surgery has become more local, so much so, that probably Morgagni himself, if he were here with us, would be amazed to find how much modern medicine differs from the old tradition, and how little resemblance it has to that of Galen. Nevertheless, this is all a development of his own thought concerning the *sedes morborum*, or, as I have termed it, anatomical thought. This thought rules modern physiology and pathology; it will certainly remain the thought of the future."

The great aim of modern medicine, as Virchow perceives, and as we must all perceive with him, is certainty. The mathematician is a satisfied man because he is certain; similarly, the engineer finds high pleasure in his work because he is certain—certain in his objects, certain in his methods, certain, even before a single stone is laid, of his ultimate results. When will the doctor be as satisfied and happy as these? Never, until his work is crowned with the same assurance of successful certainty. In the answer to the question, "*Ubi est morbus?*" —Where is the disease?—the skilled clinician can usually be as certain as the mathematician or the engineer. In the answer to the question next in the process, "*Quid est morbus?*"—What is the disease?—there is much less assurance of certainty. But in due time, even here, we may hope for definite demonstration. Then comes the question of questions, "*Quid est faciendum?*"—What is to be done, in practice, in order that the patient may be restored to health? These three questions constitute the stern, but not inglorious, tripod of modern medical difficulty and achievement. If the great International Congress at Rome had done nothing else, it would still have rendered invaluable service to practical medicine by eliciting from the venerable and philosophic Virchow so eloquent and convincing an argument on behalf of that neglected but indispensable principle of successful practice—the definite localisation of disease. The details of medicine are infinite, and to many minds bewildering, in their seeming irrelevancy. Great is the man, and worthy of all admiration, who propounds for us those two or three fundamental principles which are as feet to the lame and eyes to the blind.

